# Notes and Notices

**Published:** 1895-04

**Authors:** 


### NOTES AND NOTICES.

Dr. C. H. Krause has been elected Professor of Chemistry and Toxicology and Superintendent of the Chemical Laboratory in The Homœopathic Medical College, of Misouri. He has been acting in the capacity of Lecturer on these branches in this college for about two years.

The Alumni Association of the Hahnemann Medical College, Philadelphia, requests the pleasure of the company of the Alumni of the College at its annual reunion and banquet, on Thursday, May 2d, 1895.

The Business Meeting will convene at 4.30 p. m. in Alumni Hall, Hahnemann Medical College, Broad Street, above Race, Philadelphia, and the Banquet will be held at 10 p. m. at "The Stratford," corner of Broad and Walnut Streets.

The Trustees and Faculty of the College extend a cordial invitation to all the members of the Alumni and their friends to attend the Forty-seventh Annual Commencement, to be held on the same evening, at 8 o'clock, at the Academy of Music, Broad and Locust Streets, Philadelphia.

Banquet cards can be secured from any officer of the Association, at $3.50 each. The cards being limited to two hundred, the committee cannot guarantee to furnish any applied for after May 1st, 1895. If members wish to be present at the banquet they can secure a place by notifying the Secretary.

W. W. Van Baun, M. D., *Secretary*,
419 Pine Street, Philadelphia, Pa.

Children's Homœopathic Hospital, of Philadelphia.—The receipts of this institution for 1894 were $10,031.38, and the expenditures $9,840.88. The admissions to the wards were 184, with only six deaths. The Out-Patient Department footed up a total of 22,997 applications for relief at the Institution, while 3,263 out-door visits were made. There are 34 inmates at the present time, and there is needed more ward-room for both sick and accident cases. May I inquire whether or not you would like to join with us, and lend your aid and a helping hand in our laudable work of benevolence?

Bushrod W. James,
*President.*

N. E. Cor. 18th and Green Sts., Philadelphia.

Unfortunate.—"I was at a little mixed gathering the other evening," says Dr. E. B. Sangree, "when an incident occurred that rather brought down the house. The conversation had turned on to appendicitis, and in the course of the talk a gentleman remarked that Mr. Johnson had had his vermiform appendix removed. A deafish old lady present pricked up her ears at this, and asked, 'What was that you said?' Raising his voice, the gentleman answered, 'I said that Mr. Johnson had his vermiform appendix removed.' Very sympathetically, and in loud tones, the old lady replied, 'Oh! what a pity; and he wanted children so badly, too!' "—*Medical Arena.*

"SENNINE" is recommended where a dry dressing is indicated, and is manufactured by the Dios Chemical Company, after first having consulted Sir Joseph Lister and other authorities upon Antiseptics, and also receiving the expressed opinion of the most prominent surgeons and bacteriologists in this country as well as Europe, giving their practical experience as to the best antiseptic for dry dressing.

DR. L. D. ROGERS, editor of *The People's Health Journal*, Chicago, writes us under date of March 21st: "This is the commencement season in Chicago. Chicago Homœopathic College graduated sixty-eight students on March 19th. The Hahnemann graduates seventy-three to-day, and the National will graduate sixteen next Tuesday.

"I go on duty at Cook County Hospital April 1st.

"Statistics are being gathered for the Homœopathic Society of Chicago, which redound very much to the credit of Homœopathy. Homœopathy has shown best in Cook County in the treatment of pneumonia. The death-rate under old-school treatment has been shown to be more than twice as great as when under homœopathic treatment. Even in surgery the mortality rate under homœopathic treatment is surprisingly less than that of the old school. In diphtheria alone have the figures been unfavorable to Homœopathy."

THE AMERICAN INSTITUTE OF HOMŒOPATHY will convene at Newport, R. I., Thursday, June 20th, at three o'clock, P. M. The local committee have been and are still hard at work, and have already completed many of their arrangements. Among the plans definitely settled are:

A concert and reception, music to be by Reeve's famous American Band; an old-fashioned Rhode Island clam-bake, and a trip to Block Island, thirty miles out on the Atlantic. Add to these, the other plans of the committee and the special attractions at Newport as a noted summer resort, and there can be no question as to the excellence of the programme.

But the recreative and social side, although important, is not the most important to the Institute. The scientific side—the work done in medical science and allied subjects—this is the chief interest and duty of the Institute.

The meeting at Newport should be made noteworthy because of the scholarly value and timeliness of the papers presented, and because of thoughtful and scientific discussions.

Now is the time for each chairman to do most effective work. The prospects are that the Newport meeting will be one of the largest and most successful ever held. The interest this year seems to be general.

One more *very important matter.* Let every member of the Institute get at least *one* new member. It is a prime duty often neglected. Blanks will be furnished on application, and each member will receive one when the annual announcement is issued in May.

This circular will contain full information concerning programme of meeting, railroad fares and routes, special meetings, hotels, "The Mission," etc., and will be sent to every homœopathic physician in the United States. Let every member of the Institute now determine to attend the Newport meeting and so arrange his business that he may not be kept away.

Make the Newport meeting distinguished for a large and loyal attendance, broad, profound, and tolerant learning, and marked by such earnestness of purpose as shall give impetus and power to the school at large long after the Institute of '95 has passed into history.

EUGENE H. PORTER, A. M., M. D., *General Secretary,*
181 W. Seventy-third Street, New York City.

DR. WM. C. RICHARDSON, wife and daughters, of St. Louis, have returned home from a six weeks' vacation spent at their cottage in Ozona, Florida.

THE LATE A. J. TAFEL.—It may be of interest to our readers to know that the death of Mr. A. J. Tafel, of the firm of Boericke & Tafel, will make no difference in the business of that firm, which will be conducted by the surviving partners on the same lines as in the past. The corps of employees remains unchanged.

Dr. WM. D. GENTRY has accepted the position of Medical Director of the great National Sanitarium for Consumptives, located at Fort Union, New Mexico. He will therefore remove his office to this place. He also takes with him the type and general outfit of his new journal, *Record of the Homœopathic Materia Medica,* which was noticed in the February number of this journal at page 104.

THE Minnesota State Homœopathic Institute will hold its twenty-ninth annual session in St. Paul on Tuesday, May 29th, and continue in session three days. C. B. Pillsbury, M. D., is the President and Henry C. Aldrich, M. D., 313 Medical Block, Minneapolis, is the Secretary.

DR. NATHANIEL SCHNEIDER, of Cleveland, Ohio, died February 4th, 1895, after a long illness. Dr. Schneider was born November 1st, 1839, at Hamilton, Canada. He studied with Dr. Beckwith, of Cleveland, and graduated from the Cleveland Homœopathic Hospital College in March, 1864. He devoted himself to surgery, and for the past five years was Professor of Surgery in the Cleveland Medical College. He was for two years Vice-President of the American institute of Homœopathy.

THE AMERICAN MEDICAL PUBLISHERS' ASSOCIATION will hold its annual meeting on May 6th, in Baltimore, Md., in the parlors of the Eutaw House, at 9.30 A. M. An interesting programme is being prepared.

